# The *bandit*, a New DNA Transposon from a Hookworm—Possible Horizontal Genetic Transfer between Host and Parasite

**DOI:** 10.1371/journal.pntd.0000035

**Published:** 2007-09-27

**Authors:** Thewarach Laha, Alex Loukas, Supatra Wattanasatitarpa, Jenjira Somprakhon, Nonglack Kewgrai, Paiboon Sithithaworn, Sasithorn Kaewkes, Makedonka Mitreva, Paul J. Brindley

**Affiliations:** 1 Department of Parasitology, Faculty of Medicine, Khon Kaen University, Khon Kaen, Thailand; 2 Division of Infectious Diseases and Immunology, Queensland Institute of Medical Research, Brisbane, Queensland, Australia; 3 Genome Sequencing Center, Department of Genetics, Washington University School of Medicine, St. Louis, Missouri, United States of America; 4 Department of Tropical Medicine, Tulane University, Health Sciences Center, New Orleans, Louisiana, United States of America; University of Technology, Sydney, Australia

## Abstract

**Background:**

An enhanced understanding of the hookworm genome and its resident mobile genetic elements should facilitate understanding of the genome evolution, genome organization, possibly host-parasite co-evolution and horizontal gene transfer, and from a practical perspective, development of transposon-based transgenesis for hookworms and other parasitic nematodes.

**Methodology/Principal Findings:**

A novel *mariner*-like element (MLE) was characterized from the genome of the dog hookworm, *Ancylostoma caninum*, and termed *bandit*. The consensus sequence of the *bandit* transposon was 1,285 base pairs (bp) in length. The new transposon was flanked by perfect terminal inverted repeats of 32 nucleotides in length with a common target site duplication TA, and it encoded an open reading frame (ORF) of 342 deduced amino acid residues. Phylogenetic comparisons confirmed that the ORF encoded a *mariner*-like transposase, which included conserved catalytic domains, and that the *bandit* transposon belonged to the *cecropia* subfamily of MLEs. The phylogenetic analysis also indicated that the *Hsmar1* transposon from humans was the closest known relative of *bandit*, and that *bandit* and *Hsmar1* constituted a clade discrete from the *Tc*1 subfamily of MLEs from the nematode *Caenorhabditis elegans*. Moreover, homology models based on the crystal structure of *Mos1* from *Drosophila mauritiana* revealed closer identity in active site residues of the catalytic domain including Ser281, Lys289 and Asp293 between *bandit* and *Hsmar1* than between *Mos1* and either *bandit* or *Hsmar1*. The entire *bandit* ORF was amplified from genomic DNA and a fragment of the *bandit* ORF was amplified from RNA, indicating that this transposon is actively transcribed in hookworms.

**Conclusions/Significance:**

A *mariner*-like transposon termed *bandit* has colonized the genome of the hookworm *A. caninum*. Although MLEs exhibit a broad host range, and are identified in other nematodes, the closest phylogenetic relative of *bandit* is the *Hsmar1* element of humans. This surprising finding suggests that *bandit* was transferred horizontally between hookworm parasites and their mammalian hosts.

## Author Summary

Because of its importance to public health, the hookworm parasite has become the focus of increased research over the past decade—research that will ultimately decipher its genetic code. We now report a gene from hookworm chromosomes known as a transposon. Transposons are genes that can move around in the genome and even between genomes of different species. We named the hookworm transposon *bandit* because hookworms are “thieves” that steal the blood of their hosts, leading to protein deficiency anemia. The *bandit* transposon is a close relative of a well studied assemblage of transposons, the *mariner*-like elements, known from the chromosomes of many other organisms. The founding member of this group—the *mariner* transposon—was isolated originally from a fruit fly; *mariner* has been harnessed in the laboratory as a valuable gene therapy tool. Likewise, it may be feasible to employ the *bandit* transposon for genetic manipulation of hookworms and functional genomics to investigate the importance of hookworm genes as new intervention targets. Finally, *bandit* may have transferred horizontally from primates to hookworm or vice versa in the relatively recent evolutionary history of the hookworm–human host–parasite relationship.

## Introduction

Almost one billion people throughout tropical and sub-tropical latitudes are infected with hookworms. In the countries affected, hookworm infection is often the major contributor to iron-deficiency anemia, a direct consequence of the parasite's blood-feeding activities [1]. Comparatively little is known about the genome or population genetics of hookworms. The karyotype of only one hookworm species, the dog hookworm, *Ancylostoma caninum*, is known where the haploid chromosome number *n* = 6 [2]. Hookworms are dioecious and sex determination is by an XX-XO mechanism as in their free-living relative, the model nematode *Caenorhabditis elegans* [3]. Although the genome size of hookworms has not been reported, it may be of similar dimensions and complexity to that of *C. elegans*-around 100 megabase pairs (Mb) and containing about 20,000 protein-encoding genes (see [3]). By contrast, flow cytometric based techniques have shown that the haploid genome size of two trichostrongyle nematodes, *Haemonchus contortus* and *Teladorsagia circumcincta*, is ∼50 Mb in length [4]. Trichostrongyle nematodes are more closely related to hookworms than is the free-living nematode, *C. elegans* [5].

Over 20,000 expressed sequence tags (ESTs) from *A. caninum* and the related parasite, *A. ceylanicum*, have been characterized to some degree [6–8], including transcripts from the gut of adult worms[9]. Interestingly, most of the genes share homologues in *C. elegans*, highlighting the suitability of this free-living nematode as a model for hookworm developmental biology [8]. Moreover, the Genome Survey Sequences (GSS) Database at GenBank contains nearly 100,000 genome survey sequences from *A. caninum* (http://www.ncbi.nlm.nih.gov/dbGSS/dbGSS_summary.html), which when assembled provide a 57.6 Mb unique sequence, establishing a tractable framework for an eventual genome sequence. It can be anticipated that an enhanced understanding of the hookworm genome will aid in the control of hookworm disease and hookworm-associated anemia, including the development of new anti-parasite interventions [10].

A substantial proportion of the genome of most metazoans is composed of repetitive sequences, including various types of mobile genetic elements (MGEs). MGEs are drivers of genome evolution [11]. In addition to this role, from a practical perspective MGEs offer potential as transgenesis and gene silencing vectors [12–14], technologies that have yet to be reliably established for the study of parasitic nematodes. Problematically, however, their interspersed, repetitive nature can impede progress during genome sequencing using shotgun sequencing approaches through the confounding effects of their repetitions on sequence assembly algorithms [15,16]. For these and other reasons, knowledge of hookworm MGEs is of theoretical and practical value. Recently we reported the presence of a family of non-long terminal repeat (LTR) retrotransposons, the *dingo* retrotransposons, from the genome of *A. caninum* [17]. Here we report the presence of a *mariner* like transposon, termed *bandit*, within the genome of *A. caninum*. *Bandit* is a DD(34)D family *mariner*-like transposon [18] which, intriguingly, is much more closely related to the human *mariner*-like element *Hsmar1* than to any other MLE so far reported from other species of the phylum Nematoda.

## Methods

### Genomic DNA of the hookworm *Ancylostoma caninum*

Adult *A. caninum* hookworms were collected from naturally infected dogs from Ta Rae district, Sakonnakorn province, Thailand, as described previously [17]. After removal from the canine small intestines, the hookworms were identified microscopically as *A. caninum*, and the living worms were snap frozen and stored at −80°C. Subsequently, genomic DNA (gDNA) of adult mixed sexes of *A. caninum* was isolated from the parasites using a Qiagen genomic tip-100/G column and genomic buffer set kit (Qiagen, Germany) according to the manufacturer's instructions. Briefly, worms (50–100 mg) were lysed in DNase-free lysis buffer supplemented with RNase (Qiagen) using a DNase-free glass homogenizer. Proteinase K was added to the extracts and incubated at 50°C for 2 hours. The homogenate was clarified by centrifugation, the supernatant applied to a Qiagen genomic-tip column (Qiagen), the eluted *A. caninum* gDNA recovered by ethanol precipitation, dissolved in TE buffer, and its concentration and purity determined using a spectrophotomer.

### Construction and screening of hookworm genomic DNA libraries; bioinformatics

Size selected plasmid libraries of gDNA from adult *A. caninum* were constructed as described [17]. Briefly, gDNA was digested with the endonuclease *Hin*d III and *Xba* I (Fermentas, Sweden) and size separated through 0.8% agarose gel. Fragments ranging in size from 2–7 kilobase pairs (kb) were excised, eluted from the gel, and ligated into plasmid pBluescript SK (+/−) (Stratagene). Bacterial *E. coli* strain XL-1 blue cells were transformed with the ligation products and recombinant colonies selected by blue-white screen on LB agar supplemented with ampicillin. White colonies were transferred to wells of 96-well microtitre plates and cryopreserved in 20% glycerol at −80°C.

**Figure 1:**
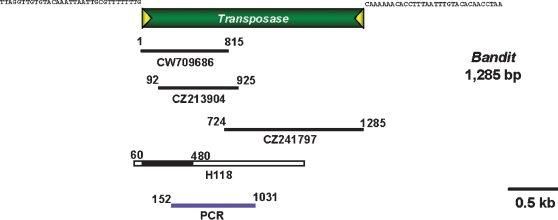
Schematic diagram representing clones and genomic survey sequences (GSS) from public databases which were used to assemble the consensus sequence of the *bandit* transposon from the genome of *Ancylostoma caninum.* Numbers on clones represent the nucleotide positions within the consensus, full length *bandit* sequence. GenBank accession numbers of contributing GSS clones are provided. The sequences of the terminal inverted repeats are presented in the top panel. In clone H118, the black colored region is bandit sequence whereas the white region on non-bandit encoding DNA.

Mobile genetic element (MGE)-like gene fragments were identified from dbEST using text and blast searches. MGE fragments were amplified by polymerase chain reaction (PCR) from gDNA and used to probe gDNA libraries (see below). At the outset, a gene probe was obtained by PCR using primers AcCR1F (5′-CAATTCTCCGATAAGGCAATG) and AcCR1R (5′-CGCGTATCCCATAGAATGTCA) specific for an *A. caninum* transcript annotated in GenBank to have identity to reverse transcriptase (GenBank AW700339), with PCR cycling conditions of 35 cycles of 94°C for 1 min, 55°C for 1 min and 72°C for 1.5 mins, and a final elongation step at 72°C for 10 mins. An amplicon encoding a retrotransposon-like gene was sequenced to confirm its identity, and the probe was named *AcCR1* (not shown). Subsequently, a transposon-like gene probe (genomic DNA clone H118; GenBank DQ377715) was obtained by library screening with *AcCR1*. Nucleotides 118–416 of the insert of H118 were PCR amplified, and after labeling with digoxygenin (DIG), the PCR product was employed to screen ∼500 clones from the size selected, *Hin*d III and *Xba* I libraries of *A. caninum* gDNA. The inserts of positive clones were sequenced and the sequences used to search the non-redundant database of GenBank using the Blastn, Blastx and tBlastx algorithms [19]. Genomic DNA and cDNA of *A. caninum* were amplified with the aim of obtaining longer fragments of the *A. caninum* transposon, using specific primers, AcMarinerF; 5′-GCTCACTCTTGGCTTGGTTC and AcMarinerR; 5′-TAATCGATTGGCGAAAGGTC, spanning nucleotide residues 154 to 1,033 of the consensus sequence of the full-length *bandit* transposon (Figure 1). PCR conditions were 94°C for 1 min, 55°C for 1 min and 72°C for 3 min, 35 cycles after which PCR products were ligated into plasmid pTOPO (Stratagene) and sequenced.

A consensus sequence of a new transposon was assembled from the positive clones and also from *A. caninum* GSS sequences in GenBank with assistance from the contig assembly program of BioEdit version 7.0.5.2 [20] (Figure 1). To identify *bandit*-like sequences in related hookworm species, the *bandit* transposase (342 amino acids) was queried against 4,953 polypeptides from *A. ceylanicum* [8] and 2,328 polypeptides from *N. americanus* [21]. Only the best homologous sequence is reported, including the identity and similarity values for the longest high-scoring segment pair (HSP) in each subject.

### Southern hybridization analysis

Thirty μg of *A. caninum* gDNA were cleaved with the restriction enzymes, *Xho* I and *Xba* I. The *bandit* probe sequence did not include recognition sites for either of these enzymes. Digested gDNA was fractionated by electrophoresis through 0.8% agarose gel, after which the fragments were transferred to nylon membrane (Hybond-N+, Amersham Biosciences) by capillary action. The *bandit*-specific probe was obtained by PCR using specific primer AcMarinerF; 5′-GCTCACTCTTGGCTTGGTTC and AcMarinerR; 5′-TAATCGATTGGCGAAAGGTC, spanning nucleotide residues 154 to 1,033 of the consensus sequence of the full-length *bandit* transposon (Figure 1). Southern hybridization analysis was performed using DIG labelled probes and detection system (Roche, USA). The membranes were incubated in hybridization medium under high stringency conditions. High stringency washing conditions were performed as recommended by the manufacturer. Signal was detected by exposure to X-ray film (Fuji).

### Reverse transcription-PCR

Total RNA of *A. caninum* mixed sex adult worms was extracted using the Nucleospin RNA II kit (Machery-Nagel, Germany) according to the manufacturer's procedures. RT-PCR was performed using the RobusT II RT-PCR Kit (FINNZYMES, Finland), primers P118F (5′-CTTCTAACGGATAGCTGCGGA and P118R (5′-GGGCGCTCTCTGATCCATCTT) specific for the *bandit* transposase based on the sequence of genomic clone H118 (GenBank accession number DQ377715) spanning nt. 118–417 (Figure 1), and the following PCR cycling conditions: 42°C for 30 mins and 94°C for 2 mins for the first cycle, 94°C for 1 min, 55°C for 1 min and 72°C for 1.5 mins, for 40 cycles, and finally an elongation step at 72°C for 10 mins. RT-PCR products were sized by electrophoresis through a 1% agarose gel. To confirm the identity of the RT-PCR products, they were transferred to nylon membranes [22], and probed with a DIG-labelled *bandit* probe (residues 152 to 1031 of *bandit*, shown in Figure 1). Southern hybridization analysis was performed using DIG labelled probes and the DIG detection system from Roche. Signals were detected by exposure to X-ray film (Fuji).

### Phylogenetic analysis

The entire transposase ORFs of *bandit* and other related elements were employed for construction of the phylogenetic tree. Alignments of amino acid sequences of functional domains were accomplished with ClustalW [23] and edited with Bioedit version 5.0.9 [20]. Sequence alignments for phylogenetic analysis comparing the conserved transposase domains were adjusted as described previously [24,25]. A phylogenetic analysis was performed on this sequence alignment using PROTDIST in PHYLIP packages and a tree was constructed using the neighbor joining method (PHYLIP, version 3.6 software) [26]. A distance matrix analysis was also carried out using maximum parsimony. The resulting phylogenetic trees were displayed using TreeView [27]. Statistical significance of branching points was evaluated with 1,000 repetitions in a bootstrap analysis (SEQBOOT). The predicted protein sequences were obtained directly from the GenBank entries where provided, otherwise ORFs were predicted by translating the nucleotide sequences provided in GenBank.

### Homology modeling

The transposase ORFs of *bandit* and *Hsmar1* were used as a query for the Swiss-Model comparative protein modeling server (http://swissmodel.expasy.org). Homologues of known structure were sought from the Research Collaboratory for Structural Bioinformatics (RCSB) Protein Data Bank (http://www.rcsb.org./pdb/home/home.do). Models were viewed and manipulated in first approach mode using Swiss-PdbViewer (http://swissmodel.expasy.org/spdbv).

## Results

### A *marine*r-like transposon present in the genome of *A. caninum*

**Figure 2:**
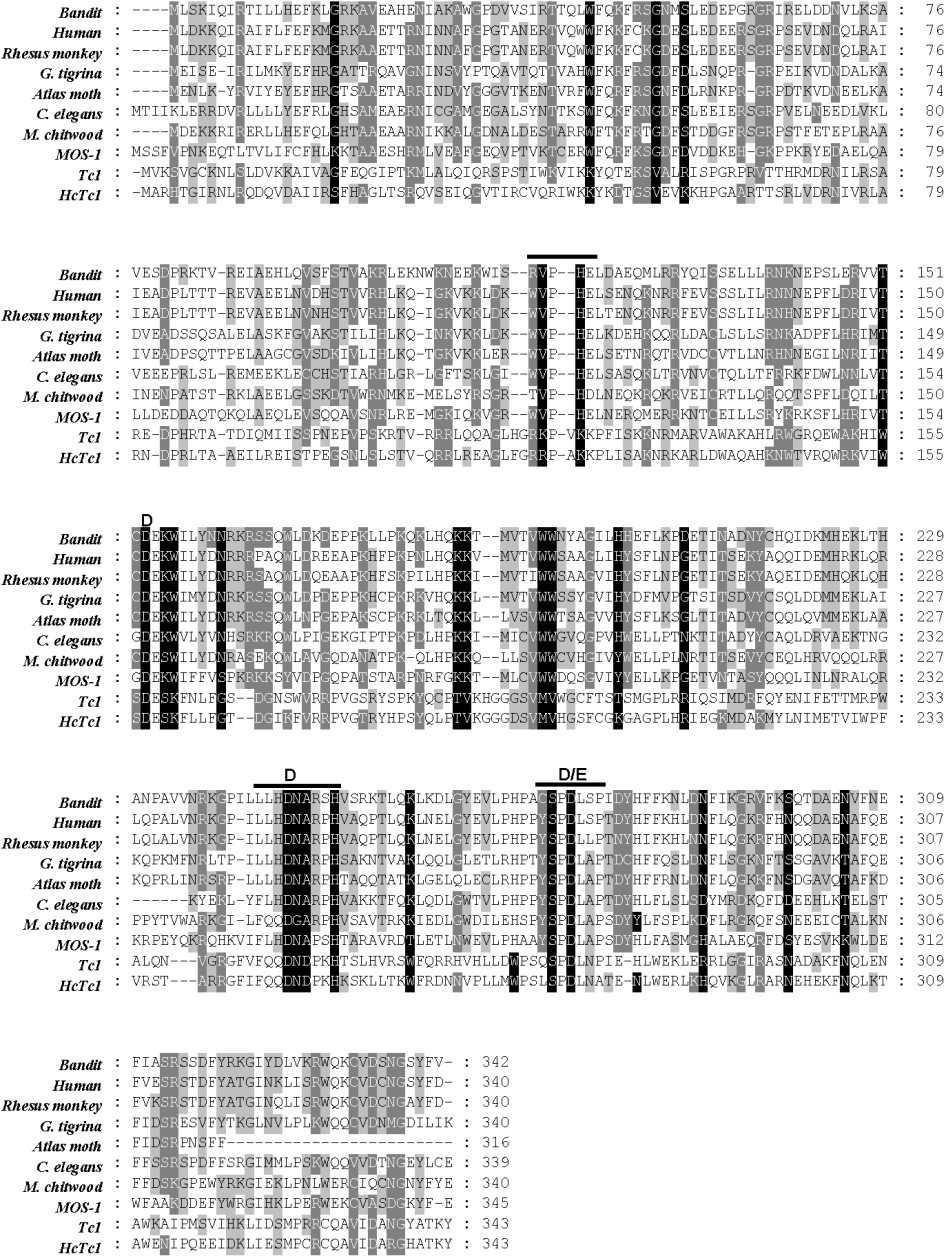
Multiple sequence alignment of the transposases of *bandit* with those from related transposons. The position of the catalytic triad domain DD(34)D/E is indicated. The conserved motifs of mariner-like elements were overlined. Conservation of residues is indicated by the shading of boxes. The GenBank accession numbers of these aligned transposons are human (Hsmar1, AAC52010), Rhesus monkey (XP_001099426), *G. tigrina* (CAA50801), Atlas moth (BAA21826), *C. elegans* (T23086), *Meloidogyne chitwoodi* (CAD26968), MOS-1 (AAC16609), *Tc1* (P03939), *HcTc1* (AAD34306).

A positive clone was identified from an *A. caninum* genomic DNA library that was screened with a reverse transcriptase-like gene probe, clone H118 (GenBank accession number DQ377715). The clone showed sequence identity with *mariner*-like transposons from many eukaryotes including *mariner* from *Homo sapiens* and *mariner* from *Bos taurus*. Sequence analysis revealed that clone H118 contained sequence that encoded part of a transposase protein (Figure 1). The consensus full length transposon was constructed using clone H118 and multiple GSSs identified by homology searches from the GenBank database (GenBank accession numbers CW709686, CZ213904 and CZ241797) (Figure 1). We termed the new transposon *bandit*, in keeping with the informal convention of naming mobile genetic elements with terms suggestive of a peripatetic lifestyle (e.g. *mariner*, *hobo* and *fugitive*)[28–30]. Given the present results, the name *bandit* seemed appropriate since a bandit is often difficult to apprehend, and in this present context, it appears that *bandit* has moved furtively between hookworms and their mammalian hosts (see below). The consensus sequence of *bandit* was 1,285 bp flanked by 32 nt perfect terminal inverted repeats at each extremity with a common target site duplication TA (Figure 1 and Figure S1). *bandit* has one ORF of 342 amino acid residues encoding for a transposase enzyme. The *bandit* transposase contained the conserved DD34D motif that is found in the active site of the catalytic C-terminal domain of *mariner*-like transposons as opposed to the DDE motif found in the Tc1-like elements [12] (Figure 2). The ORF of the *bandit* showed highest similarities to *Hsmar1* from human (55% identity, 70% similarity), *Bos taurus* (54% identity, 70% similarity) and *Tc1* of *C. elegans* (41% identity, 58% similarity), *HcTc*1 of *Haemonchus contortus* (22% identity, 42% similarity). On the other hand, no *bandit*-like sequences were identified in the National Center for Biotechnology Information (NCBI) catalogue of dog sequences (not shown), indicating that bandit is not of canine origin.

The perfect inverted repeats of 32 bp are the standard length for *mariner*-like elements [31] compared with 54 bp for Tc1 from *C. elegans* [32] and 55 bp for *HcTc*1 from *H. contortus* [33]. In addition to the catalytic triad, *bandit* contains most of the additional canonical features of *mariner*-like elements (MLEs); the WVPHEL motif (RVPHEL in *bandit*) and YSPDLAP (CSPDLSP in *bandit*) [34]. However, *bandit* did not contain the conserved FLHDNARPH motif that overlaps the second D of catalytic triad in most MLE transposases. In *bandit*, this motif is replaced by a LLHDNARSH motif [35,36] (Figure S1).

### Numerous copies of *bandit* interspersed throughout the *A. caninum* genome

**Figure 3:**
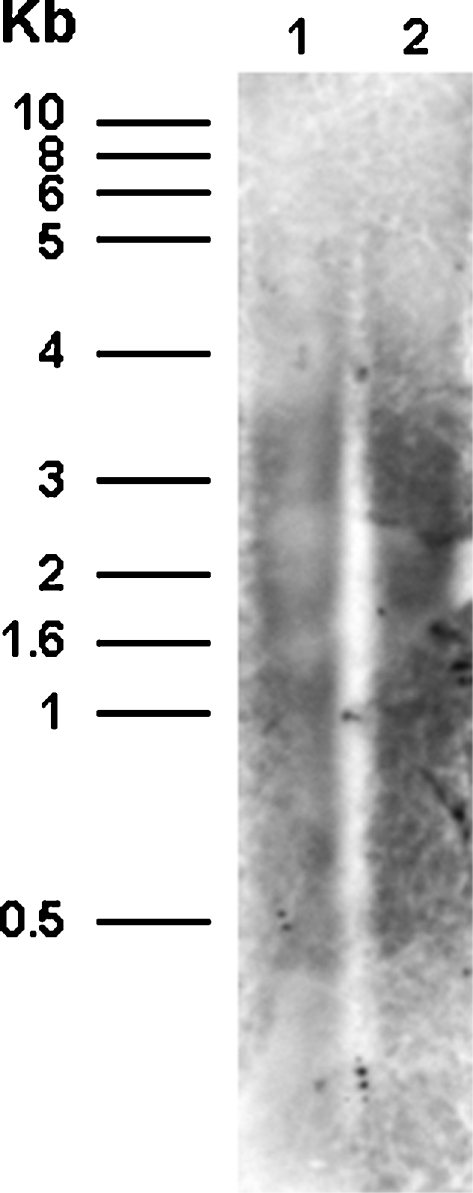
Southern hybridization analysis of *Ancylostoma caninum* genomic DNA to a probe specific for the *bandit* transposon. The genomic DNAs were cleaved with endonucleases *Xba* I (lane 1) and *Xho* I (lane 2). Molecular size standards in kilobase pairs (kb) are shown at the left.

Smeared bands of hybridization were evident when a Southern blot of *A. caninum* genomic DNA (gDNA) was probed with the labeled *bandit*-specific sequence. *Xba* I and *Xho* I were used to cleave the gDNA, and hybridization of each restriction digest to a *bandit*-specific probe revealed a smear-like pattern of numerous bands of hybridization ranging in size from >5-<0.5 kb (Figure 3), confirming the presence of numerous copies of the *bandit* transposon in the genome of natural populations of *A. caninum* from north-eastern Thailand. This also suggests that the *bandit* element is widely dispersed in the hookworm genome rather than being localized at just one or a few isolated sites. To more specifically address the copy number, we queried the *A. caninum* GSS in NCBI with the *bandit* sequence using blastn and tblastx algorithms. Using blastn, we identified 23 GSS with 87–98% identity over at least 250 bp. Using tblastx, we identified >200 GSS with >90% identity over at least 50 amino acids (not shown). The *A. caninum* GSS are predicted to cover about 15% of the genome (M. Mitreva, unpublished). Extrapolating from these numbers there may be between 150–1,500 copies of bandit dispersed throughout the genome.

### *bandit* is a novel *mariner*-like transposon of the cecropia subfamily

**Figure 4:**
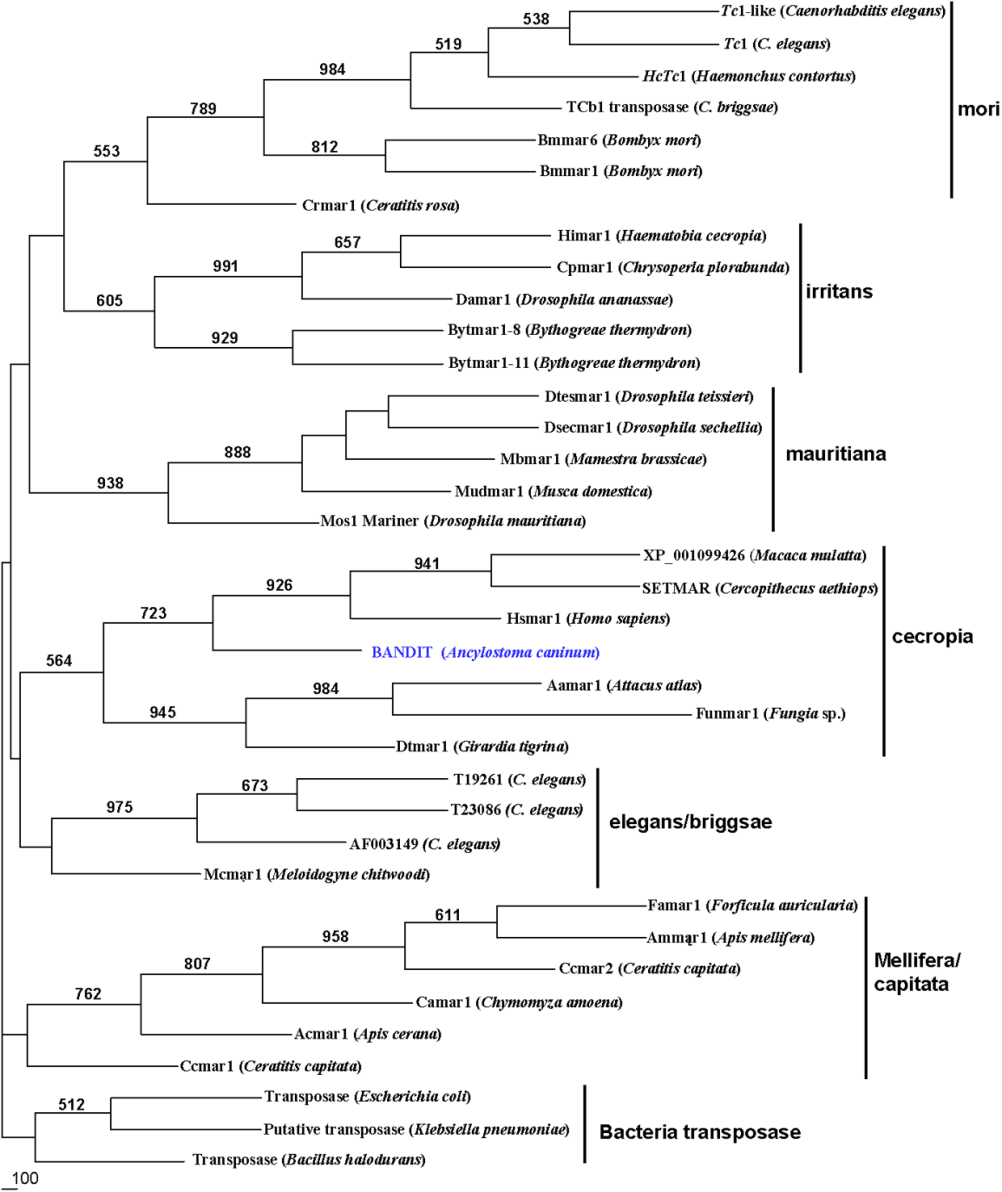
Phylogram constructed using the neighbor-joining method to compare the relationships among transposases of the *bandit* transposon and of representative mariner-like elements belonging to the *Tc1/mariner* superfamily of transposons from a range of host genomes. Representatives of six clades of mariner-like elements including the mori, irritans, mauritiana, and cecropia were included in the analysis. The elements used in the tree includes Tc1-like (AAD12818) and Tc1 (P03934), T19261, T23086 and AF003149 from *C. elegans*, HcTc1 (AAD34306) from *Haemonchus contortus*, TCb1 (CAA30681) from *C. briggsae*, Bmmar1 (U47917) and BmMar6 (AAN06610) from *Bombyx mori*, Crmar1 (AAK61417) from *Ceratitis rosa*, Himar1 (ABB59013) mutagenesis vector pFNLTP16H3, Cpmar1 (AAC46945) from *Chrysoperla plorabunda*, Damar1 (DAU11648) from *Drosophila ananassae*, Bytmar1-8 (CAD45868) and Bytmar1-11 (CAD45369) from *Bythogreae thermydron*, Dtesmar1 (AAC28261) from *D. teissieri*, Dsecmar1 (AAC16609) from *D. sechellia*, Mbmar1 (AAL69970) from *Mamesta brassicae*, Mudmar1 (AK54758) from *Musca domestica*, Mos1 (pdb2F7T) from *D. mauritiana*, XP_001099426 from *Macaca mulatta*, SETMAR (ABC72092) from *Cercopithecus aethiops*, Hsmar1 (AAC52010) from *Homo sapiens*, Aamar1 (BAA21826) from *Attacus atlas*, Funmar1 (BAB32436) from Fungia sp., Dtmar1 (CAA50801) from *Girardia tigrina*, Mcmar1 (CAD26968) from *Meloidogyne chitwoodi*, Famar1 (AAO12863) from *Forficula auricularia*, Ammar1 (AAO12861) from *Apis mellifera*, Ccmar2 (AAO12864) from *Ceratitis capitata*, Camar1 (AAO12862) from *Chymomyza amoena*, Acmar1 (BAB86288) from *Apis cerana*, Ccmar1 (AAB17945) from *Ceratitis capitata*. The outgroup included transposases from gram positive and negative bacteria including *Bacillus halodurans* (BAA75315), *Escherichia coli* (AAB28848) and *Klebsiella pneumoniae* (CAB82575). Bootstrap values, where 500 or greater from a maximum of 1,000 replicates, are presented at the nodes.

A phylogenetic tree was constructed based on the sequence alignment of the entire transposase ORFs of *bandit* and 37 other transposon sequences available in public databases. A neighbor-joining tree with 1,000 replicates revealed that *bandit* is most closely related to *Hsmar1* from *Homo sapiens* (Figure 4). *Mariner*-like transposons can be classified into six subfamilies [24,25]. *Bandit* formed a clade with elements from the cecropia subfamily with solid bootstrap support (564), and this diphyletic clade included a branch containing *bandit* and three primate-originated MLEs, and a branch with *Funmar*1 from the coral *Fungia* sp., *Aamar*1 from the atlas moth, *Attacus atlas* and *Dtmar*1 from the planarian, *Girardia tigrina*. The appearance of the branches of the cecropia clade was the same when either neighbor joining or maximum parsimony (not shown) methods were employed in tree construction. Indeed, bootstrap support for the clade that included bandit and the primate elements was even stronger in the maximum parsimony analysis (982) than that obtained using the neighbor joining method (723). The phylogenetic distance between human and hookworm is far greater than that reflected in the phylogenetic analysis of these transposons, suggesting to us that *bandit* is only distantly related to MLEs from nematodes that are closely related to *A. caninum*, and is much more similar to transposons from the hookworm's mammalian hosts. For example, the MLE HcTc1 from the trichostrongyle parasite, *H. contortus* (a close relative of *A. caninum*) belongs to the *mori* clade of MLEs (Figure 4). The remarkable identity between *bandit* and the primate MLEs, *Hsmar1* and SETMAR, strongly suggests horizontal transmission of this element from host to parasite (or vice versa).

### Homology models confirm close identity of hookworm *bandit* and human *Hsmar1* transposons

**Figure 5:**
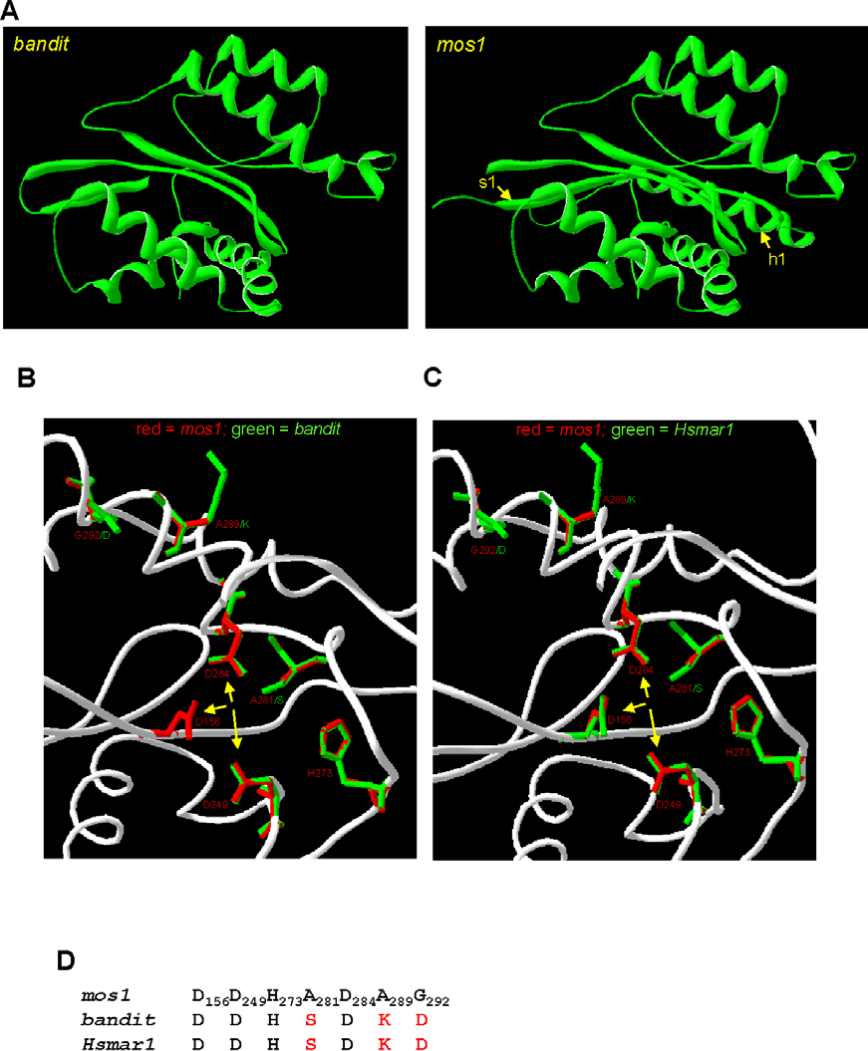
Homology models of *bandit* from *Ancylostoma caninum* and *Hsmar1* from *Homo sapiens* based on the crystal structure of the *mos1* element from *Drosophila mauritiana.* Ribbon diagram showing the predicted structure of the catalytic domains of *bandit* and *mos1* (A). s1 and h1 refer to β sheet number 1 and α helix number 1 of the *mos1* catalytic domain–homologous regions were present in *bandit* but were not included in the model. Superimposition of the catalytic active sites of *bandit* (B) and *Hsmar1* (C) on the crystal structure of *mos1* highlighting the residues involved in catalysis. Conserved active site residues are labeled in red font; where bandit or Hsmar1 active site residues differ from mos1, the substitution is denoted in green font. Yellow arrows denote the three catalytic Asp residues. Numbering of side chains is based on the *mos1* sequence. Comparison of the residues predicted to be involved in catalysis from *bandit*, *Hsmar1* and *mos1* (D). Residues selected were based on the crystal structure of *mos1*.

The catalytic C-terminal domain of the predicted transpose ORF of *bandit* was modeled on the crystal structure of the C-terminal catalytic domain (residues 126–345) of *mos1* transposase from *Drosophila mauritiana* (pdb accession number 2f7tA). The structural alignment spanned residues 158–345 of *mos1* and 178–342 of *bandit*. The general fold of the *bandit* catalytic domain was highly conserved with that of *mos1* (Figure 5A). The first alpha helix and beta sheet of the catalytic domain of *bandit* (including the first catalytic Asp residue) were too dissimilar to *mos1* to be included in the model; however, the rest of the domain revealed similar active site architecture. Because *bandit* is most similar to human *Hsmar1* at the primary sequence level (Figure 4), we also modeled the catalytic domain of *Hsmar1* transposase on the crystal structure of *mos1*. The sequence conservation between *mos1* and *Hsmar1* also was high (Figure 2). Surprisingly, when the key active site residues of the catalytic domains [37] of *bandit* and *Hsmar1* were compared with those of *mos1*, we observed that *bandit* and *Hsmar1* had identical active site residues but, by contrast, three of these residues had non-conservative substitutions in *mos1* (Figure 5B, C and D).

### *bandit* is transcribed in the parasitic stages of *A. caninum*

**Figure 6:**
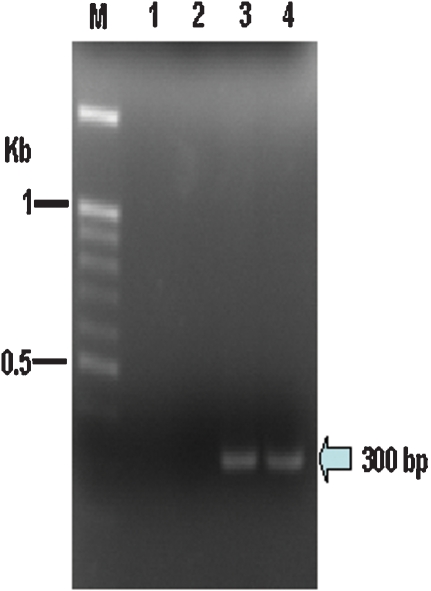
Reverse transcription PCR targeting the *bandit* transposon. Transcripts encoding the transposase of *bandit* were amplified by PCR from cDNA of the adult mixed sex of *A. caninum*. Products of the expected size, 300 bp are indicated with the arrow; lane 1, negative control where reverse transcriptase was omitted from the reaction; lane 2, empty lane; lane 3, plasmid DNA of clone H118 (positive control); lane 4, cDNA of mixed sex adult hookworms. Molecular size standards (lane M) are shown at the left.

Transcripts encoding the transposase of *bandit* were amplified by PCR from cDNA from mixed sex adult hookworms. Products of the expected size, 300 bp, were amplified (Figure 6), and the identity of the amplicons was confirmed by sequence analysis and Southern hybridization using a *bandit*-specific probe (not shown). Together with the presence of relatively intact inverted repeats, this approach indicated that functional domains of the element are transcribed in the adult hookworm, and suggests that copies of *bandit* are active and mobile within the genome of *A. caninum*.

### *bandit* integrates into non-coding regions of the *A. caninum* genome

**Figure 7:**
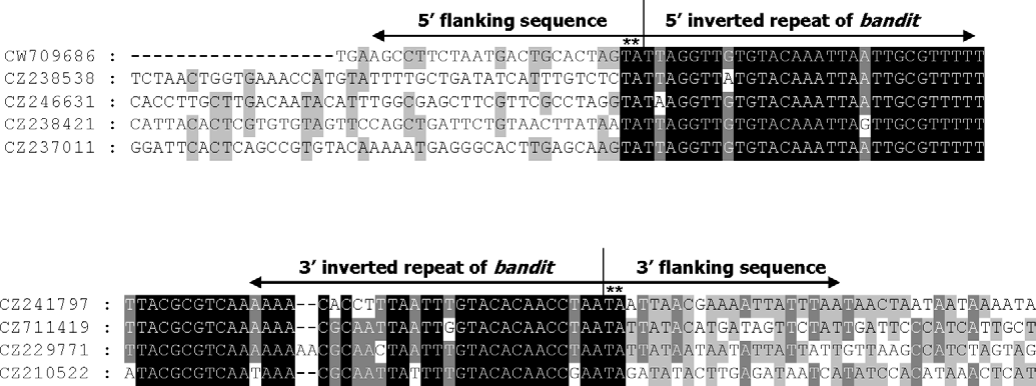
Multiple sequence alignment of the nucleotide sequences flanking the insertion sites of copies of the *bandit* transposons within the genome of *Ancylostoma caninum.* Alignments of nucleotide sequences flanking the 5′- (A) and 3′- (B) termini of *bandit*. Conservation of residues is indicated by the shading of boxes. Target sequences, with GenBank accession numbers as indicated on the left, were identified among entries in the GSS database of *A. caninum* sequences at GenBank. The target site TA duplications are indicated with asterisks.

Sequences flanking the different individual copies of *bandit* (from the GSS dataset) were aligned (Figure 7). Blast search analysis of the 5′ and 3′ flanking regions of *bandit* did not show homology to sequences in the public database. The flanking DNA was however generally AT-rich and appeared to be of non-coding origin.

### *bandit* in related hookworm species

Available transcriptomic data of related hookworm species, *A. ceylanicum* [8] and *N. americanus* [21] was explored to identify putative *bandit*-like transposons. The similarity search (BlastX) resulted in identification of a homologous sequence from *A. ceylanicum* (contig id AE04671, 44% identity, 64% similarity over 185 amino acids) and from *Necator americanus* (contig id NAC01255, 45% identity, 58% similarity over 91 amino acids) (data not shown). Based on these interspecific partial matches the conservation is lower compared to *A. caninum bandit* and *Hsmar 1* (55% identity, 70% similarity), but higher between the *A. caninum* bandit and other hookworm bandit-like sequences than with the *HcTc1* from the ruminant blood-feeder *H. contortus* (22% identity, 42% similarity) or the *Tc1* from *C. elegans* (41% identity, 58% similarity). Unavailability of the full length ORF of the *bandit* from these two related hookworm species contributed to their exclusion from the above described analysis.

## Discussion

A new member of the Tc1/*mariner* superfamily of DNA transposons has been characterized from the genome of a parasitic nematode, and termed *bandit*. Sequence identity, structure, and phylogenetic relationships demonstrated that the *bandit* transposon belonged to the *cecropia* sub-family of *mariner*-like elements (MLEs). The cecropia clade is populated by transposons from diverse animal taxa including the cecropia moth [38], a coral [39], primates including the African green monkey and humans [40] and now from a hookworm. Earlier reports dealing with members of this clade have suggested that horizontal transmission has likely been involved in the present disposition of its members (e.g., [38]). In like fashion, given that the closest relatives of *bandit* are *Hsmar1* and SETMAR from humans and monkeys, *bandit* may have been transmitted to or from hookworms and their primate hosts.

The *bandit* transposon displayed the structural hallmarks of the *Tc1*/*mariner* superfamily of transposons including an overall length of ∼1.3 kb, a single ORF encoding a transposase of 342 amino acid residues in length, a DD(34)D catalytic motif, duplication of TA dinucleotide pairs upon insertion and inverted terminal repeats of 32 bp in length [12]. The DD(34)D motif indicated that *bandit* was a *mariner*-rather than a *Tc1*-family member. Phylogenetic analysis confirmed that *bandit* was indeed *mariner*-like and, remarkably, indicated that its closest relative was the primate *Hsmar1* transposon. Moreover, homology models established using the crystal structure coordinates of *mos1* transposase (from *D. mauritiana*) revealed closer identity between *bandit* and *Hsmar1* than between *bandit* or *Hsmar1* and *Mos1* in active site architecture and catalytic domain residues.

The hookworm, *A. caninum*, is a parasite of dogs but is frequently found in the human small intestine. Although it does not generally reach sexual maturity in humans, it may now be evolving this capacity [41]. Moreover, *A. caninum* larvae commonly infect human skin resulting in pruritic dermatitis termed cutaneous *larva migrans* [42]. *A. caninum* is closely related to the anthropophilic hookworm, *Ancylostoma duodenale*, and another close relative, *A. ceylanicum*, parasitizes both humans and dogs. (The human hookworms *A. duodenale* and *N. americanus* infect more than 700 million people, causing widespread morbidity–primarily iron deficiency anemia– and mortality [1]). The intimacy of host-parasite relationships is known to facilitate horizontal transmission of genetic material [43], and parasitism is known to facilitate horizontal transmission of transposons. For example, *P* elements have been transferred among *Drosophila* species by a parasitic mite [44], as have *mariner*-like elements between parasitic wasps and their lepidopteran hosts [45]. Since the closest known relative of *bandit* is *Hsmar1* from humans, and given the parasitic association between hookworms and primates–the hosts of *bandit* and *Hsmar1*, respectively–it is likely that the presence of *bandit* and *Hsmar1* in both parasite and host genomes reflects parasitism-facilitated horizontal transmission.

After entry into a naïve lineage, an active autonomous MLE undergoes unrestrained spread through transposition and sexual exchange for a time until regulatory and/or mutational inactivation dampens transposition activity and associated deleterious mutations [46,47]. Given that transcription of *bandit* was detected by RT-PCR analysis, and given that the intact integration footprint of *bandit* within the hookworm genome remains readily apparent, it appears that *bandit* is transpositionally active within the *A. caninum* genome. If so, the hypothesized horizontal transmission of *Hsmar1/bandit* elements between host and parasite may be a recent event, and since *Hsmar1* is now inactive [48], the direction of the horizontal transfer may have been from host to parasite.

Eukaryotic genomes generally include substantial amounts of sequences derived from MGEs, primarily retrotransposons and transposons. These mobile sequences are drivers of genome evolution [11]. A number of MGEs have been characterized from nematode genomes including *Tas*, a LTR retrotransposon, and *R4*, a non-LTR retrotransposon, both from *Ascaris lumbricoides* [49,50], *mariner*-like elements (MLEs) from *Trichostrongylus colubriformis* [51] and the *RTE1*, *NeSL*, and *Cer* retrotransposons from *C. elegans*. Recently, it was reported that the *A. caninum* genome includes elements with identity to the *Transib* superfamily of transposons. In vertebrates, the *Transib* transposon has mutated to form the RAG1 protein and recombination signal sequences involved in catalyzing B and T cell receptor gene V(D)J recombination [52]. Also, recently we described the *dingo* non-LTR retrotransposons from the genome of *A. caninum* [17] and numerous transcripts encoding reverse transcriptase are evident in the EST database of *A. caninum*, *A. ceylanicum* and *N. americanus* hookworms (http://nematode.net), indicating the presence of endogenous retroviruses or retrotransposons. Based on the genomes of *C. elegans* [53] and several parasitic helminths including schistosomes [54], it is apparent that that the hookworm genome has been colonized not only by the *bandit* transposon, but also by numerous other waves of MGEs. From a practical perspective, understanding of MGE complexity, diversity and copy numbers can be expected to facilitate the assembly and annotation of the hookworm genome sequence (a focus of current genome sequencing effort, http://nematode.net). Finally, as with other MGEs, an endogenous hookworm *mariner*-like transposon such as *bandit* holds potential as a transgenesis vector for manipulation of the hookworm genome, given the ability of other *Tc1*/*mariner* superfamily members such as *mos1* to transpose within the genomes of *C. elegans*, planarians and other species (e.g., [55–57]).

## Supporting Information

Figure S1 Consensus nucleotide and deduced amino acid sequence of the entire *bandit* element. Sequence features of the *bandit* are indicated within duplicated TA dinucleotides. The inverted repeats at both ends are highlighted with green. The ORF starts at the Met encoded at nt. 189 and terminates at the stop codon at nt. 117, encoding an enzyme of 342 amino acid residues. Two conserved hallmark motifs of *mariner*-like elements [38] are highlighted with grey and the catalytic triad DD34D residues are indicated by red colored font.
(0.03 MB DOC)
