# Supplementary material for: The bandit, a New DNA Transposon from a Hookworm—Possible Horizontal Genetic Transfer between Host and Parasite
Source: PLoS Negl Trop Dis. 2007 Sep 27;1(1):e35. doi: 10.1371/journal.pntd.0000035 (PMC2041818; doi:10.1371/journal.pntd.0000035)
Supplement: Figure S1 — Consensus nucleotide and deduced amino acid sequence of the entire bandit element. Sequence features of the bandit are indicated within duplicated TA dinucleotides. The inverted repeats at both ends are highlighted with green. The ORF starts at the Met encoded at nt. 189 and terminates at the stop codon at nt. 117, encoding an enzyme of 342 amino acid residues. Two conserved hallmark motifs of mariner-like elements [38] are highlighted with grey and the catalytic triad DD34D residues are indicated by red colored font. (0.03 MB DOC) [file pntd.0000035.s001.doc]

1 TA**TTAGGTTGTGTACAAATTAATTGCGTTTTTTTG**CAAACCAGTTTTTGACCCACTGGTT 60

61 TTCGTGTATTGTTCATGTAACGTTCCCAATGTTGTACACCATTTAGAAGCTTATCTTTCG 120

121 CTCATTCATTTCGTGATGTTCATTTGTTTCGAAGCTCACTCTTGGCTTGGTTCCGCCCGT 180

181 TATTCAAGATGCTCTCCAAGATACAAATTCGTACCATTTTGCTTCATGAGTTCAAGCTTG 240
    M L S K I Q I R T I L L H E F K L G

241 GACGGAAAGCAGTGGAGGCTCACGAAAATATCGCGAAAGCATGGGGACCAGATGTCGTTT 300
    R K A V E A H E N I A K A W G P D V V S

301 CAATTCGCACCACTCAACTCTGGTTCCAAAAATTCCGATCAGGAAACATGAGCCTTGAAG 360
    I R T T Q L W F Q K F R S G N M S L E D

361 ATGAACCAGGTCGAGGTCGCATCCGTGAACTTGACGACAACGTCCTGAAGTCTGCGGTGG 420
    E P G R G R I R E L D D N V L K S A V E

421 AGTCGGATCCACGAAAAACTGTTCGTGAGATAGCGGAACATCTTCAAGTATCATTTTCCA 480
    S D P R K T V R E I A E H L Q V S F S T

481 CCGTCGCCAAGCGTTTGGAAAAAAATTGGAAAAATGAAGAAAAATGGATCAGTAGAGTGC 540
    V A K R L E K N W K N E E K W I S R V P

541 CACATGAATTGGACGCTGAACAGATGCTCAGGCGTTATCAGATCTCATCAGAGCTGCTTT 600
    H E L D A E Q M L R R Y Q I S S E L L L

601 TACGGAATAAAAACGAGCCTTCCCTTGAACGAGTCGTAACCTGCGACGAGAAATGGATTC 660
    R N K N E P S L E R V V T C D E K W I L

661 TCTACAATAATCGGAAACGTTCTTCGCAGTGGTTAGACAAAGACGAGCCCCCAAAACTTC 720
    Y N N R K R S S Q W L D K D E P P K L L

721 TTCCGAAGCAGAAGTTGCACCAAAAGAAGACTATGGTCACAGTTTGGTGGAATTACGCAG 780
    P K Q K L H Q K K T M V T V W W N Y A G

781 GAATTTTACATCACGAGTTCCTAAAACCAGATGAAACCATCAATGCGGACAACTACTGCC 840
    I L H H E F L K P D E T I N A D N Y C H

841 ACCAAATAGACAAAATGCATGAGAAACTGACACATGCGAATCCAGCAGTGGTGAACAGAA 900
    Q I D K M H E K L T H A N P A V V N R K

901 AAGGTCCGATTCTTCTTCTCCACGATAATGCCAGATCTCATGTTTCAAGAAAAACGCTGC 960
    G P I L L L H D N A R S H V S R K T L Q

961 AGAAGTTAAAAGACCTTGGCTACGAAGTACTGCCTCATCCGGCATGCTCCCCAGACCTTT 1020
    K L K D L G Y E V L P H P A C S P D L S

1021 CGCCAATCGATTATCACTTCTTCAAGAACCTCGACAACTTCATCAAGGGAAGAGTATTCA 1080
    P I D Y H F F K N L D N F I K G R V F K

1081 AAAGTCAGACCGATGCTGAAAATGTCTTCAACGAGTTCATAGCCTCCAGAAGTTCGGACT 1140
    S Q T D A E N V F N E F I A S R S S D F

1141 TCTACCGCAAGGGAATTTATGATCTTGTGAAACGTTGGCAAAAATGCGTAGATTCTAATG 1200
    Y R K G I Y D L V K R W Q K C V D S N G

1201 GTTCTTATTTTGTCTAATAAACTTCGCCGAGTTGTAAACGTTTAAAGTTACGCGT**CAAAA** 1260
    S Y F V *

1261 **AACACCTTTAATTTGTACACAACCTAA**TA 1289
